# A Review of Testosterone Pellets in the Treatment of Hypogonadism

**DOI:** 10.1007/s11930-014-0033-7

**Published:** 2014-10-03

**Authors:** Andrew McCullough

**Affiliations:** 23 Hackett Blvd, Albany, NY 12208 USA

**Keywords:** Hypogonadism, Testosterone pellets, Aromatase inhibitor

## Abstract

Currently, the most popular form of testosterone replacement is the topical gels that require daily applications and incur a risk of transfer of testosterone to partners and family. One of the problems with testosterone replacement is the short half-life of testosterone. A long-acting formulation is appealing to patients and physicians. In 1972, fused crystalline testosterone pellets were approved in the USA by the FDA but they were not marketed until 2008. Pharmacokinetics studies were available on a different formulation from which much can be learned and applied to the current formulation, Testopel®. The decay kinetics, pituitary suppression, and effect on other sex steroids are reviewed as well as the short-term complication rates. This review should provide the testosterone pellet implanter a better understanding of the physiology of testosterone pellet supplementation for hypogonadism.

## Introduction

The benefits of testosterone replacement for the treatment of hypogonadism are well documented. As men age, there is a steady decline in testosterone due to an aging pituitary-gonadal  axis. Current treatment modalities require repeated testosterone injections or topical application of gels. Long-term topical and injection therapy are fraught with poor long-term compliance due to the inconvenience of the application and vacillating serum levels. An ideal therapy would be one that is easy to administer, provides reliable levels, and is affordable.

Long-lasting testosterone (T) pellets were FDA-approved in 1972. At that time, the only other options available were inexpensive generic intramuscular T injections. Testopel® which was approved by the FDA is crystalline T, formulated in 75-mg pellets (3 × 8 mm) with a surface area of 98 mm^2^. The pellets are surgically placed in the subcutaneous space and gradually dissolve. Their long-lasting effect is presumed to be due to the gradual dissolution of the pellets in the relatively hypovascular subdermal space [1]. The formulation lay “dormant” until 2008 when the patent was purchased by a newly formed company, Slate. The T pellets were named Testopel®. T replacement therapy evolved between 1972 and 2008. With increased therapeutic formulations, incessant direct-to-consumer advertising, and the increasing number of males of the baby boom generation (born circa 1946–1964) seeking “eternal youth,” T replacement therapy had evolved into a multibillion-dollar pharmaceutical industry.

### Gels Versus Pellets

Gel-based T replacements have since become the preferred method of therapy since their introduction. The noninvasive nature of gels made it an appealing alternative to T injections. Yet, the need for daily applications, erratic absorption, low long-term compliance rates, the risk of T transfer to family members, and the expense of the monthly prescriptions opened the window for a “new” form of T therapy. Testopel® was thus a welcome new addition to the armamentarium as it required a simple procedure three to four times a year and delivered eugonadal T levels for periods from 3 to 6 months. Day-to-day compliance was not an issue and as such, the therapeutic efficacy was more easily assessed. Unfortunately, the prescribing information, a reflection of the regulatory standards of 1972, was lacking in specific information as to the mechanism of dissolution, the recommended dosing, the insertion technique, the pharmacokinetics, the dosing recommendations, and the data on the dosing frequency. Bioavailability studies were deferred with the original FDA application [2]. Information in the package insert was based on observations and extrapolations from data on injectable T propionate, a short-acting T ester that is no longer used. It is unclear whether any pharmacokinetic studies were ever done on the actual Testopel® pellets. There are certainly no published studies prior to 2009 on the currently FDA-approved Testopel® pellets.

### Pellet Pharmacokinetics

Much of the data on T pellet pharmacokinetics comes from work from Handelsman in Australia. The only peer-reviewed publications on T pellets are based on a pellet formulation that has never been approved in the USA produced by Organon. Nonetheless, much can be learned about the pharmacokinetics of T pellets by a critical review of those studies. In 1988, Handelsman conducted a randomized crossover comparator study in 15 previously treated hypogonadal men (9 primary and 6 secondary), using the Organon product of T pellets (6 × 100 mg). Resulting hormonal levels were the primary endpoints. This formulation is more in line with the currently used Testopel® pellets in terms of pellet surface area and T dose. The comparator arms were injectable T esters (250 mg every 2 weeks) and oral Tundecanoate (not available in the USA). The hormone levels after injectable T were assessed weekly for 1 month whereas in the T pellets levels were measured weekly for 1 month then monthly thereafter until the levels returned to baseline. Levels on the pellets peaked at 781 ng/dl at 3 weeks and returned to baseline (247 ng/dl) by week 20. Extrapolating from the data presented in the paper, a level of 300 ng/dl was reached at around 13 weeks. Serum chemistries and hematologic parameters were unchanged throughout the study. Both the pellet and injection group reported consistent subjective improvement in libido, potency, muscular strength, and general well-being. With equal numbers (six) of pellet and injection subjects, patients opted to stay on their respective therapies. Reflecting the early experience with the pellets, 6 of 15 men (40 %) extruded at least one pellet though no wound infections occurred. Six 100-mg pellets were found to maintain T levels for up to 4 months [3].

In 1990, Handelsmen published an open-label crossover pharmacokinetic study in 43 men (22 hypergonadotropic and 21 hypogonadotropic) on the three regimens (6 × 100 mg, 6 × 200 mg, and 3 × 200 mg), measuring T levels at monthly intervals. The treatments were crossed over at 6 months. The surface areas were 200 and 100 mm^2^ for the 200- and 100-mg pellet, respectively. Pellets were inserted into the sub dermal fat in the lower abdominal wall at the level of the umbilicus. Each pellet was inserted into its own tract 5–10 cm from the insertion site. He found that T levels correlated highly with the dose inserted. The levels in men with 1200 mg were nearly twice that of the men with a 600-mg insertion. Rates of absorption of T from the pellets were constant with a “near linear zero-order release of T over months that was not influenced by the size or number of pellets. The estimated half-time of absorption was approximately 2.5 months and the rate of T release was 1.3 mg/day for the 200 mg pellet and 0.65 mg/day for the 100 mg pellet.” Despite a constant absorption of T from the pellets, serum T levels peaked at 1 month and decreased monthly thereafter, supportive of first-order decay kinetics. This would suggest that as the pellets dissolve, the surface area decreases and the amount of T delivered decreases. Ninety percent of T is metabolized by hepatic CYP3A4, with approximately 10 and 1 % metabolized by 5 alpha reductase and aromatase inhibitor, respectively. As the *K*_m_ of the CYP3A4 metabolism is 50,000 nanomoles, the metabolism of T is physiologically impossible to saturate [4]. The kinetics of T decay must therefore be all attributable to the dissolution of the pellets in the subcutaneous space. Based on some intact extruded pellets and assuming that the extruded pellets had comparable dissolution properties to intact in situ pellets, he extrapolated a dissolution rate of 1.5 mg/day and maintenance of eugonadal ranges for 4–5 months. He calculated a therapeutic half-life of 2.5 months. As production rate of endogenous T is 3–9 mg/day [5], 400–1200 mg of pellets could produce eugonadal ranges for 4–6 months. LH levels, measured only in the primary hypogonadal men only, were markedly and uniformly suppressed for 1–4 months. Their levels inversely mirrored declining T levels. The reproducible effect of the pellets on gonadotropins was the basis for him recommending using them to augment clinical monitoring. SHBG levels were not affected. He reported a 10 % (10/111) extrusion rate. The frequency of pellet extrusion fell dramatically with increased experience from an incidence of 40 % after the first 15 procedures to 5 % in the later 96 implants. Some palpable fibrosis at the insertion site in some men was seen long after the pellets were dissolved. Seventy percent (30/43) expressed a desire to continue with the pellets versus injections [6].

In 1996, Jockenhovel published a comprehensive pharmacokinetic study in 14 profoundly hypogonadal men (baseline T of 34 ng/dl) using the 6 × 200 mg formulation. Only three of the men had idiopathic secondary hypogonadism. Measuring T levels nine times in the first 48 h, a burst release of T was seen with a *C*_max_ of 144 ng/dl. T levels stabilized for 63 days following which first order decay occurred. Estradiol levels peaked at 42 days, at 38 pgm/ml. At 180 days, the mean serum T was 300. Both LH and FSH exponentially decreased after insertion, inversely mirroring Tand estradiol levels. An interesting observation was the volume of distribution increased while T_1/2_ decreased with increasing BMI. Their mean BMI was less than 25.kg/m^2^. This observation is not surprising as T in the periphery equilibrates quickly between most organs and the blood [7]. Men with larger BMIs have, in general, a larger volume of distribution. When considering pellets in obese patients, they might need significantly more pellets. Despite a 5 % extrusion rate, all but one of the men expressed the desire to continue with the pellets [8].

### Complications, Insertion Technique, and Dosing

In a retrospective survey, Handelsman determined that extrusions were increased by early post implantation increased physical activity [9]. Experiencing an incidence of extrusions (5–12 %) and infections (1.4–6.8 %), he set about to determine if complication rates were affected by the technique, site of insertion, washing of the pellets, impregnation of the pellets with antibiotics, and experience of the implanter. Using the anterior abdominal wall insertion technique, he found that none of the aforementioned variables influenced extrusion or infection rates. Whereas extrusions associated with infections usually occur at a median of 4 weeks, extrusions without apparent infections occurred at 9 weeks [10, 11].

In 2004, Handelsman reviewed his experience in 136 men with a standard dose of 800 mg (4 × 200 mg pellets). Pellets were placed under the skin of the lateral abdominal wall or the lateral aspects of the buttocks along the pants line. Re-implantations were based on symptoms alone without any reminders to the patients. His extrusion rate was approximately 10 %. T levels were drawn at the implantation visit (median 299 vs baseline of 144). If extrusions occurred, the men were instructed to keep the pellets and make a note of the day of the extrusion. The pellets were desiccated upon return to the clinic and weighed to determine the amount that was left. No quantitative measurement was made of the remaining T in the pellet. A linear regression curve was generated by the desiccated weight and the day of extrusion, and a pellet dissolution rate of 1.31 mg/day was calculated. Only intact pellets were used and the pellets maintained their cylindrical shape for a median of 98 days. As nadir levels were unpredictably lowered by increased number of extrusions, one might question the strength of his assumptions. Is an extruded pellet biologically equivalent to one in situ? Handelsman suggested that a dose four pellets of 200 mg should last 5.8 months. The questions remain as to the relevance of these observations to the current Testopel® preparation. Are the formulations truly bioequivalent? Does pellet fragmentation occur during insertion? Are the decay curves the same? How does BMI affect the peak serum levels and decay curve? Should symptoms or T levels be used as replacement criteria?

### Studies and Clinical Experience

The literature on Testopel® implantation was nonexistent until the 2008 introduction of the product in the US market. In 2009, Cavender et al. published a single-site retrospective review of his experience with the 75-mg Testopel® pellets in 80 men (272 insertions) treated for clinical hypogonadism (T < 350 ng/dl). The series was uncontrolled and retrospective and with variable follow-up and treatments. It was not intended as a pharmacokinetic study but a report of a clinical experience and represented the first published reported clinical experience with Testopel®. Dosing of the pellets was arbitrarily based on severity of symptoms, body weight, age, and lifetstyle. The insertion technique was a modification of the Handelsmen lateral jackknife position and utilizeda proprietary trocar. Reported infection rates and extrusion rates were considerably lower than Handelsman at 0.3 and 0.3 % respectively, reflecting the different formulation, technique, experience of implanter, patient selection, or a combination of all the factors. Although he demonstrated normalization of T levels, because of the variability of the patients, treatments, and inclusion criteria, little could be concluded from the paper other than Testopel® could be expected to produce results at least similar tothe Organon product and that it appeared to be at least as well tolerated[12•].

Kaminetsky published an industry-supported, FDA-approved, pharmacokinetic study in 30 men with Testopel® [13••]. Dosing was based on BMI and baseline T levels and the insertion technique that was published by Cavender. Twenty-eight men received treatment (8 pellets, *n* = 3; 10 pellets, *n* = 14; 12 pellets, *n* = 12). None met the criteria for six pellets (baseline T of <315 ng/dl and a BMI of <18). Peak T levels at 1 month were dose dependent with 100, 100, 86, 75, and 14 % above 315 ng/dl at weeks 1, 4, 12, 20, and 24, respectively. The continuation phase of the study in (22 of 28 men) revealed that 100 and 31.8 had levels >315 ng/dl at week 4 and 16 after treatment. This would suggest that biologic variability of treatment effect might be expected from one treatment to the next for unexplained reasons. Previous studies have not investigated the reproducibility of the levels from one insertion to the next. During phase 1 of the study, erectile function scores increased clinically significantly in the first 12 weeks of treatment though the score returned to baseline at the end of the study. Though clinically significant, there was no placebo arm. As in other studies, pituitary gonadotropins were suppressed as T and estradiol levels increased. Unlike the Handelsman experience, no extrusions or infections were reported.

The findings in the Kaminetsky study though reassuring about the safety and efficacy of the Testopel® was in stark contrast to the FDA-approved 1972 package insert provided with the product, recommending 150–450 mg (two to six pellets) every 3–6 months. None of the studies thus reported results with such low dosing. In an attempt to provide some clarity McCullough et al. published an independent multi-institutional study on 380 patients with 702 insertions at 6 institutions. This represented the early experience at each institution. Investigators pooled their data on pre-insertion and post-insertion T levels along with the number of implanted pellets. The number of pellets to be inserted and the techniques used were based on the clinical experience of each investigator though all but one started with six pellets. Unlike the Kaminetsky study, pre-treatment level of T or BMI were not used as a criteria for the number of pellets required. With multiple investigators using their own criteria for treatment and follow-up, this represented a more generalizable guide for the new implanter. Though the T levels at 4 weeks were comparable for all pellet levels, patient and investigator disenchantment with subsequent levels lead investigators to insert more than the minimum number of pellets. Six or seven pellets were utilized in only 10 % of the insertions with 10 or more pellets being inserted in 63 %. The insertion of 10 or more pellets resulted in eugonadal levels for a longer period of time. Regardless of the number inserted, all men were hypogonadal at 6 months with most men requiring re-implantation after 4 months, much like the 1988 Handelsman study with the Organon 100-mg pellets. T levels appeared to decay exponentially behaving like first-order decay kinetics. Unlike in the Handelsman study with 200-mg pellets, inserting more pellets did not increase the period of time between insertions consistent with an exponential decay [14••].

In 2012, Pastuszak et al. reported their follow-up of 273 men, many of whom were in the original multi-institutional study. The indications for treatment were clinical hypogonadism. Mean initial T level was 328 (208) ng/dl and BMI 30 (5). Sixty-eight men had been diagnosed with prostate cancer. Based on observed levels determined at various time points, decay curves were calculated. Unlike the Jockenhovel pharmacokinetic study, many of the conclusions drawn were from extrapolated data and not from actual measured levels. This retrospective observational study examined the effect of initial T level, BMI, multiple insertions, and the number of pellets inserted. Like the multi-institutional study, adequate numbers in the 6–7-pellet group were lacking. Ninety-five percent of the men were treated with 10 or more pellets. The authors found that early post-insertion T levels were impacted by the number of pellets inserted. Higher levels were achieved with more pellets. As endogenous T production is virtually shut down with exogenous replacement, the initial T level did not impact subsequent T levels. There is therefore no need to titrate the number of pellets based on the initial T level as was done in the Kaminetsky pharmacokinetic trial. Men with BMIs less than 25 achieved higher levels than those with a BMI greater than 25, and the decay is faster. This supports the concept that volume of distribution affects subsequent hormone levels, as demonstrated by Jockhovel. Regardless of the number of insertions, number of pellets implanted, initial T level, or BMI, most men needed re-implantation between 3 and 4 months after the insertion. No men experienced a significant increase in the hematocrit or hemoglobin and no men with documented prostate cancer experienced progression for their disease. No men developed prostate cancer during the period of observation. Extrusion and infection rate were 1.1 and 0.4 % respectively, suggesting inherent differences in the nature of the pellets [15••].

### Patient Experience and Satisfaction

There have been several single-center papers addressing treatment satisfaction and compliance with Testopel®. Khera et al. investigated a small series of young men (4) with Klinefelters who would be requiring lifetime T replacement. Compliance with the Testopel® formulation was better than gels or injections [16]. Liphultz looked at patient satisfaction rates at Baylor via a survey based on patient recall. The choice of therapy was heavily influenced by physician recommendation with 53, 31, and 17 % choosing injections, gels, and Testopel®, respectively. Overall, approximately 70 % of patients were satisfied with their respective treatments regardless of modality used. Though the pellets were favored because of the ease and convenience of use, injections were favored because of their decreased cost [17•]. Regional differences in insurance reimbursement can clearly impact patient preferences. Placebo-controlled studies on patient reported outcomes are understandably lacking with Testopel®.

As with type 2 diabetes, barring major lifestyle changes, hypogonadism is a lifetime problem for which men need treatment. Clearly, T pellets offer some advantages with respect to the maintenance of consistent eugonadal levels of T. There are unfortunately no long-term studies of the use of T pellets. As with any surgical procedure, with repeated insertions, the cumulative risk of an extrusion, hematoma, or infection increases. Anecdotally, many implanters have found that with repeated insertions, subcutaneous fibrosis occurs, making the insertions more difficult. In an effort to increase the interval between insertions, McCullough et al. combined anastrozole, an aromatase inhibitor with Testopel®. The theory was that if the metabolism of Testopel® could be slowed down, T levels might remain eugonadal for a longer period of time and the interval between insertions increased. Thirty-eight men with 65 insertions were analyzed. T levels at up at 120 days were comparable between the groups. With the addition of anastrozole, T levels were maintained at eugonadal levels for over 120 days. Mean re-insertion time was increased from 124 (22) to 194 (62) days. Twenty-five percent did not require a re-insertion. The mechanism though had nothing to do with a decrease in T metabolism but was secondary to the inhibition of the suppressive effect of T replacement on pituitary gonadotropins and endogenous production. Men on Testopel® and anastrozole did not demonstrate the secondary increase in their estradiol levels or suppression of their LH and FSH levels. The addition of a generic aromatase inhibitor significantly decreased the number of annual Testopel® insertions [18•].

## Conclusions

The use of T implants as a form of T replacement has been reported since 1938 [19]. The formulation approved by the FDA in 1972 was Testopel®. Despite a lack of information provided by the package insert, there is a wealth of information available through peer-reviewed studies on similar preparations and the product itself. Pellets provide sustained eugonadal T levels for 3–6 months. Contemporary studies suggest that the FDA recommended 3–6 pellets are inadequate for most men and that 10 pellets (750 mg) produce the most reliable levels. Post implantation levels are affected by the volume of distribution, i.e., thin men require fewer, whereas morbidly obese men might require more. Extrusion and implantation infection rates at high-volume centers with Testopel® are less than 1 %, and patient acceptance of the procedure is very high. As with all forms of replacement therapy, close monitoring of therapeutic efficacy is important. More long-term studies on clinical efficacy and safety are needed.
